# Attenuating innate immunity and facilitating β-coronavirus infection by NSP1 of SARS-CoV-2 through specific redistributing hnRNP A2/B1 cellular localization

**DOI:** 10.1038/s41392-021-00786-y

**Published:** 2021-10-26

**Authors:** Fanghang Zhou, Qianya Wan, Sheng Chen, Ying Chen, Pui-Hui Wang, Xi Yao, Ming-liang He

**Affiliations:** 1 Department of Biomedical Sciences, City University of Hong Kong, Kowloon, Hong Kong SAR, China; 2 Advanced Medical Research Institute, Cheeloo College of Medicine, Shandong University, 250012 Jinan, Shandong China; 3 CityU Shenzhen Research Institute, Nanshan, Shenzhen, China

**Keywords:** Innate immunity, Infection

**Dear Editor**,

Evidence shows the NSP1’s crucial roles of the β-coronavirus SARS-CoV-2 in promoting cellular mRNA degradation, inhibiting host cell translation, innate immunity, and inducing inflammatory cytokine storm in the pathogenesis of COVID-19.^1,2^ More interestingly, NSP1 deletion in infectious clones prevents virus infection.^3^ However, little is known how NSP1 interacts with host factors to disrupt the host’s innate immunity for facilitating virus infection and reproduction. As a (+) ssRNA virus, SARS-CoV-2 completes its life cycle in the cytosol; viral RNA processing is the key for controlling and regulating the virus reproduction and pathogenesis. The ribonucleoproteins hnRNPs are the main factors responsible for RNA processing, including RNA splicing, maturation, decay, and translation, and even innate immunity in some cases.

To facilitate viral RNA processing, hnRNPs must redistribute from nucleus to the cytoplasm. We expressed SARS-CoV-2 encoded proteins NSP1, NSP2, NSP5, ORF8, and NSP12 (FLAG-tagged at the C-terminal) in Rhabdomyosarcoma cells and examined their effects on hnRNPs’ subcellular distribution. After immunostaining with specific antibodies against hnRNP A2/B1 (also called hnRNP A2 or hnRNP B1), hnRNP D, hnRNP K, and hnRNP L proteins, surprisingly, we observed that only NSP1 specifically induced the hnRNP A2/B1 redistribution from the nucleus to the cytoplasm (Fig. 1a) but had no effect on its subfamily member hnRNP A1 (Fig. 1b). All other viral proteins (NSP2, NSP5, ORF8, and NSP12) failed to redistribute hnRNP A2/B1 and the other tested hnRNPs (Supplementary Fig. 1a–d). We further examined the effects of SARS-CoV-2 proteins on hnRNP A2/B1 protein levels. As shown in Supplementary Fig. 3b, NSP14, NSP15, and ORF8 only slightly decreased the protein level of hnRNP A2/B1, while the other NSPs (NSP1, NSP2, NSP3, NSP4, NSP5, NSP9, NSP12, and NSP13) had no obvious effects on hnRNP A2/B1 protein level in HEK293T cells.

**Fig. 1:**
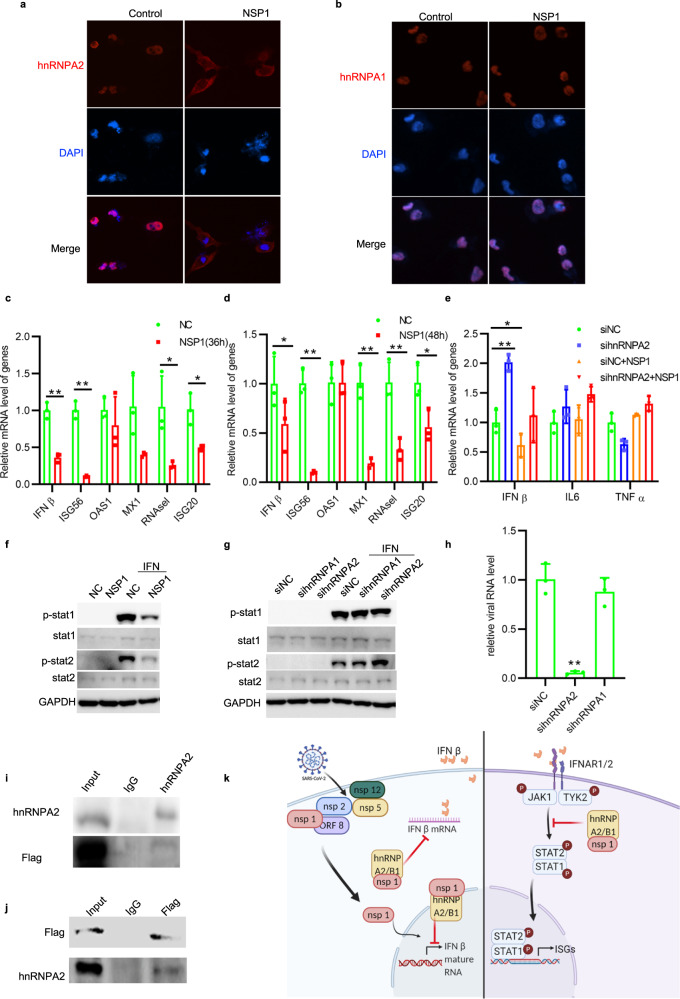
**a** RD cells were ectopically expressed NSP1. After 48 h, hnRNP A2/B1 subcellular location is observed under a fluorescence microscope. hnRNP A2/B1 is indicated as Red. Blue is DAPI. **b** RD cells were ectopically expressed NSP1 at 48 h. After 48 h, hnRNP A1 subcellular location is observed under a fluorescence microscope. hnRNP A1 is indicated as red. Blue is DAPI. **c**, **d** HEK293T cells were transfected with NSP1 for 36 or 48 h. Then mRNA level of IFNß and its downstream ISGs was detected by RT-qPCR, unpaired *t* test, *P* < 0.01. **e** 293T cells were firstly silenced hnRNP A2/B1 by specified siRNA for 24 h, following the ectopic expression of viral protein NSP1 for 48 h. The mRNA levels of IFNß, TNF alpha, and IL-6 in 293T cells were determined by qRT-PCR, unpaired *t* test, *P* < 0.05. **f** HEK293T cells were transfected with control/NSP1 plasmids for 48 h, then treated with/without IFNα (1000 U/ml) for 30 min. Cell lysates were collected for western blot assay. **g** HEK293T cells were transfected with specific siRNA for 24 h followed by 1000 U/ml of IFNα treatment for 30 min. Cell lysates were collected for western blot assay. **h** RT-qPCR result of intracellular viral RNA level in RD cells infected with HCoV-OC43 at 72 h post infection, unpaired *t* test, *P < 0.05. Cells were firstly treated with siNC, sihnRNP A2/B1, and sihnRNP A1 siRNA separately, and then infected with hCov-OC43 after 24 h. **i**, **j** 293T cells were ectopically expressed NSP1 at 48 h. Specific antibodies anti-Flag or anti-hnRNP A2/B1 were used for protein immunoprecipitation assay. Normal rabbit or mouse antibodies were used as control. **k** Scheme (created with BioRender.com.) of SARS-CoV2 NSP1 in regulating host immune response

Recent studies show that NSP1 blocks the initiation of the host mRNA translation, drives mRNAs into the decay pathway, and may impair host immunity and stimulate an inflammatory response.^1^ We thus investigated its effects on the host cell’s innate immunity and inflammatory cytokine expression, the main death causes of COVID-19 patients. Interestingly, NSP1 decreased the mRNA level of IFNβ measured by RT-qPCR assay (Fig. 1c, d). We knocked down hnRNP A2/B1 by specific siRNAs, leading to an increased mRNA level of IFNß (Fig. 1e). More importantly, the decreased IFNß mRNA level in NSP1-expressing cells was able to completely restore by silencing hnRNP A2/B1 expression (Fig. 1e). Surprisingly, the mRNA level of major inflammatory factors (e.g., TNFα and IL-6) was not affected by either hnRNP A2/B1 knockdown or the forced NSP1 expression (Fig. 1e). We then conducted two more experiments to examine whether NSP1 affects the gene expression of type I interferons’ downstream antiviral effectors—interferon-stimulated genes (ISGs). (1) With ectopic NSP1 expression, we observed that the mRNA level of ISG56, MX1, RNAase L, and ISG20 was significantly decreased (Fig. 1c, d); (2) We treated HEK 293T cells with IFNα (1000 U/ml) for 30 min and examined the phosphorylation status of STAT1 and STAT2. As shown in Fig. 1f, NSP1 decreased STAT1/2 phosphorylation (Fig. 1f); and STAT2 phosphorylation was significantly boosted by knockdown of hnRNP A2 but not hnRNP A1 upon IFNα stimulation (Fig. 1g). We further performed co-immunoprecipitation assay to determine whether hnRNP A2/B1 could interact with NSP1. We showed that NSP1 directly bonds hnRNP A2/B1 (Fig. 1i, j), implying that SARS-CoV-2 hijacks host hnRNP A2/B1 protein redistribution through direct binding with NSP1 to impair host innate immunity to facilitate SARS-CoV-2 infection.

Targeting the host factor displays particular antiviral advantages because it would not only avoid the fast viral mutagenesis but also inhibit viral reproduction and infection against a board range of virus species. We aligned the NSP1 sequences of all human pathogenic β-coronavirus species, including SARS-CoV, SARS-CoV-2, and closed related animal species, MERS-CoV, HCoV-HKU1, and HCoV-OC43. As shown in Supplementary Fig. 2, the NSP1 sequence of HCoV-OC43 shares the lowest similarity with SARS-CoV-2 as compared with other pathogenic β-coronavirus species. To prove the concept, we knocked down hnRNP A2/B1 in Rhabdomyosarcoma cells and infected them with HCoV-OC43, and measured the intracellular viral RNA level by RT-qPCR. Knockdown of hnRNP A2/B1 significantly suppressed viral replication by decreasing the viral genomic RNA level by 98%, whereas no antiviral effects were observed when its subfamily member hnRNP A1 was knocked down (Fig. 1h), demonstrating that NSP1 specifically hijacks hnRNP A2/B1’s cellular localization to suppress the host cells’ innate immunity for facilitating SARS-CoV-2 and other ß-coronaviruses (such as HCoV-OC43) infections.

The molecular mechanism on how NSP1 specifically redistributes hnRNP A2/B1 in the cytosol is to be investigated. We noticed that hnRNP A2/B1 is almost localized in the nucleus in normal cells and redistributed in the cytoplasm (Fig. 1a). We observed NSP1 distribution both in the cytoplasm and nucleus in Hela cells, which is consistent with a recent report.^2^ To exclude the possibility that the FLAG-tag affects NSP1 function or distribution, we constructed a plasmid to express the natural NSP1 of SARS-CoV-2 in HeLa cells. Both NSP1 with/without Flag-tag induced hnRNP A2 redistribution (Supplementary Fig. 1e). Results from our study revealed that NSP1 formed a complex with hnRNP A2/B1 (Fig. 1i, j). Taken together, we propose a dual mechanism for NSP1 hijacking hnRNP A2/B1 cellular redistribution (Fig. 1k). NSP1 may bind newly synthesized hnRNP A2/B1 in the cytoplasm and block its translocation into the nucleus, and the nuclear NSP1 may form a complex with hnRNP A2/B1 and other unknown proteins that drive hnRNP A2/B1 to translocate into the cytoplasm. NSP1 is reported to inhibit the initiation of translation through global ribosome runoff that may cause the redistribution of hnRNP A2/B1 in the cytoplasm. To test this possibility, we applied translation inhibitor Cycloheximide (CHX) in our study and observed that the NSP1-induced cytoplasmic hnRNP A2 distribution was no change whether the cells were treated with or without CHX (Supplementary Fig. 1f). Besides suppressing IFNß expression, NSP1/hnRNP A2 complex also disrupts type I interferon signaling by decreasing the phosphorylation level of STAT1/2. To our surprise, NSP1 did not have any effects on inflammatory cytokine transcription as it was previously proposed. Our study is well consistent with a recent report showing that NSP1 suppressed inflammasome activation.^4^ Knockdown of hnRNP A2/B1 only activated IFNß expression (Fig. 1c, d) and NSP1 only decreased the IFNß mRNA level but not inflammatory-related gene expression such as TNFα and IL-6 (Fig. 1e). NSP1 was reported to exhibit common biological functions for inducing endonucleolytic cleavage of host mRNAs.^5^ Using ribosomal 18S RNA as internal control, we showed that NPS1 indeed did not commonly decrease the mRNA levels of host genes (Supplementary Fig. 3a), demonstrating the specificity of suppressing IFNβ expression by NSP1 through binding with and redistributing hnRNP A2/B1 cytosol localization. More importantly, depletion of hnRNP A2/B1 almost completely suppressed HCoV-OC43 replication, indicating the importance of targeting hnRNP B2/A1 for treating pan β-coronavirus infections, such SARS-CoV, MERS-CoV, SARS-CoV-2, and their large number of mutants.

## Supplementary information

Attenuating Innate Immunity and Facilitating β-Coronavirus Infection by NSP1 of SARS-CoV-2 through Specific Redistributing hnRNP A2/B1 Cellular Localization

## Data Availability

The datasets used and/or analyzed during this study are available from the corresponding author on reasonable request.
