# Supplementary material for: Attenuating innate immunity and facilitating β-coronavirus infection by NSP1 of SARS-CoV-2 through specific redistributing hnRNP A2/B1 cellular localization
Source: Signal Transduct Target Ther. 2021 Oct 26;6:371. doi: 10.1038/s41392-021-00786-y (PMC8546379; doi:10.1038/s41392-021-00786-y)
Supplement: Supplementary file 1 — Attenuating Innate Immunity and Facilitating β-Coronavirus Infection by NSP1 of SARS-CoV-2 through Specific Redistributing hnRNP A2/B1 Cellular Localization [file 41392_2021_786_MOESM1_ESM.docx]

**Supplementary information**

**Attenuating Innate Immunity and Facilitating β-Coronavirus Infection by NSP1 of SARS-CoV-2 through Specific Redistributing hnRNP A2/B1** **Cellular Localization**

Fanghang Zhou^1,#^, Qianya Wan^1,#^, Sheng Chen^1,#^, Ying Chen^1^, Pui-Hui Wang^2^, Xi Yao^1*^, Ming-liang He^1, 3*^

^1^ Department of Biomedical Sciences, City University of Hong Kong, Kowloon, Hong Kong SAR, China.

^2^ Key Laboratory for Experimental Teratology of Ministry of Education and Advanced Medical Research Institute, Cheeloo College of Medicine, Shandong University, Jinan, Shandong, 250012, China.

^3^ CityU Shenzhen Research Institute, Nanshan, Shenzhen, China

*Correspondence Contact Information

Address: 1A-202, 2/F, Block 1, To Yuen Building

Phone: +852 3442-4492

Fax: +852 3442-0549

Email: [mlhe7788@gmail.com](mailto:mlhe7788@gmail.com) or [xi.yao@cityu.edu.hk](mailto:xi.yao@cityu.edu.hk)

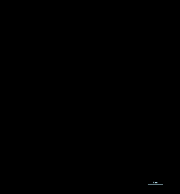

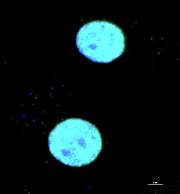

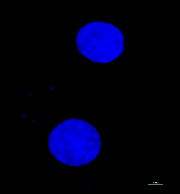

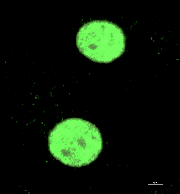

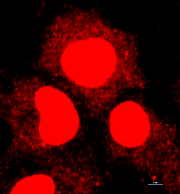

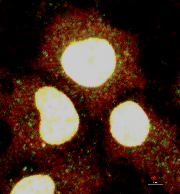

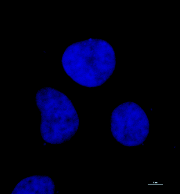

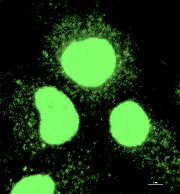

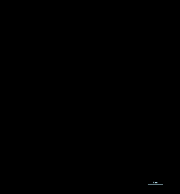

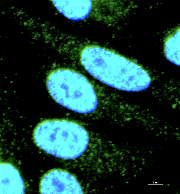

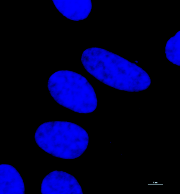

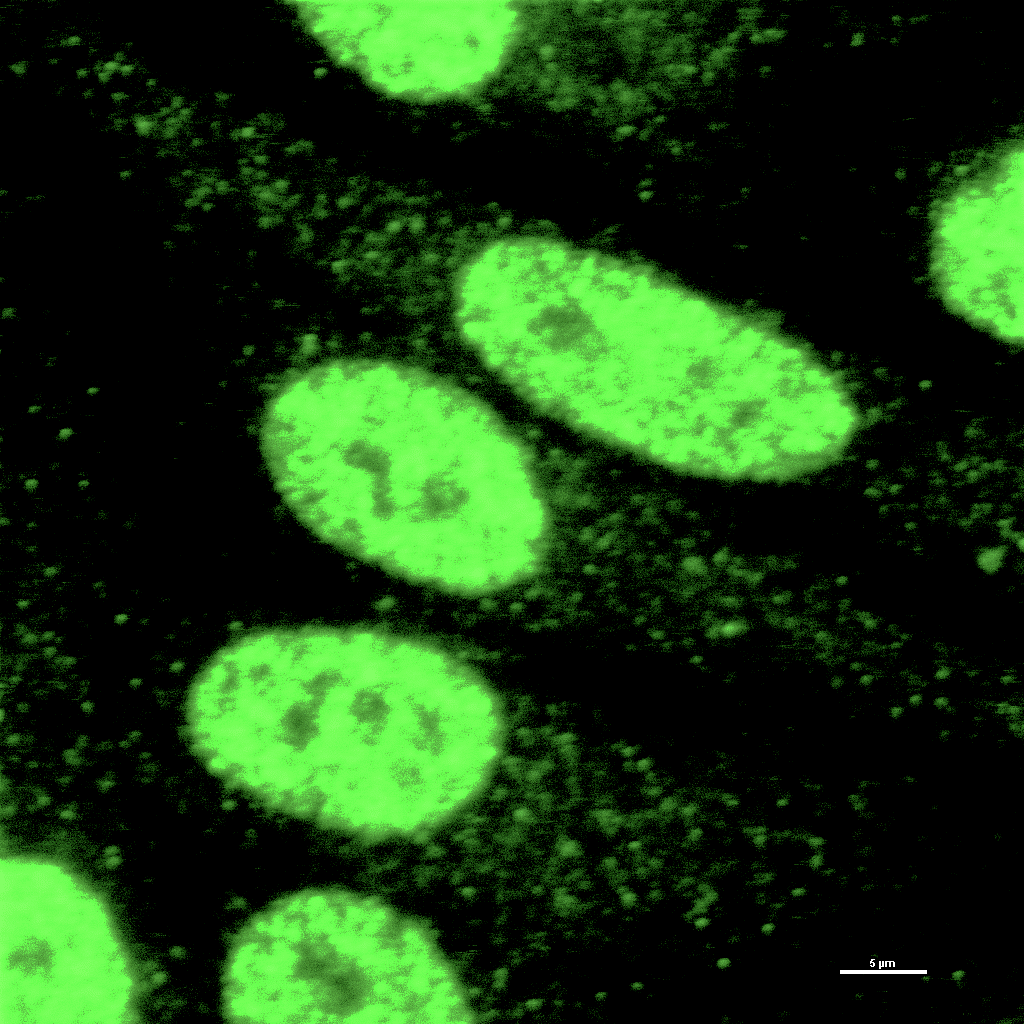


NC

NSP1-Flag

NSP1

hnRNPA2

Flag

DAPI

Merge

e

NSP1

NC

NC+

CHX


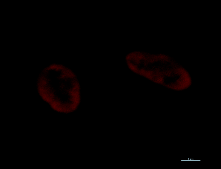

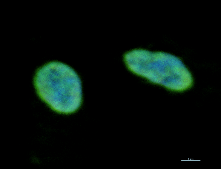

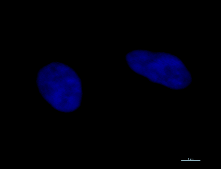

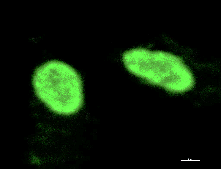

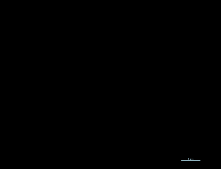

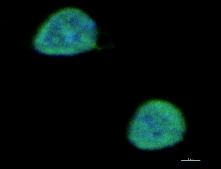

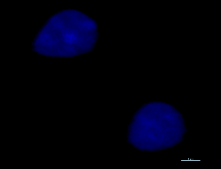

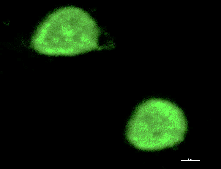

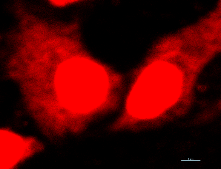

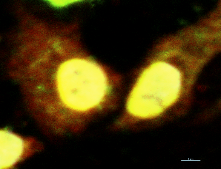

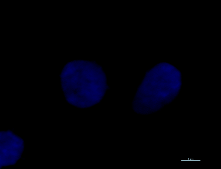

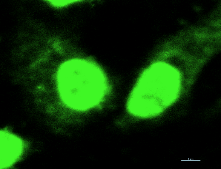

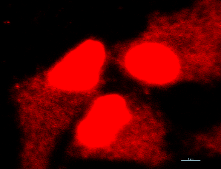

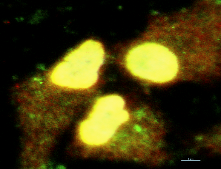

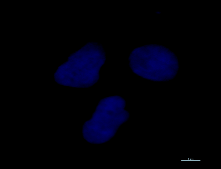

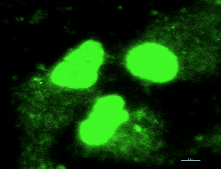


NSP1+

CHX

hnRNPA2

Flag

DAPI

Merge

f

**Supplementary Fig.1 Screening for the functions of hnRNP family proteins with SARS-CoV-2 viral proteins ectopically expression. a-d** RD cells were transfected with plasmids for expression of SARS-CoV-2 proteins NSP1, NSP2, NSP5, NSP12 and ORF8. After 48 hours, the cellular location of hnRNP A2, K, D and L is observed under fluorescence microscope. hnRNP family proteins are stained with red color, the cell nucleus is stained with DAPI (blue).**e.** Hela cells were ectopically expressed NSP1. After 36 hour, hnRNP A2 subcellular location is observed under fluorescence microscope. hnRNP A2 is indicated as Green. NSP1-Flag is indicated as Red. Blue is DAPI. **f**.Hela cells were transfected with NC/NSP1 for 30h, then cells were treated with 50ug/ml CHX for 6h. The cellular location of hnRNP A2 is observed under fluorescence microscope. HnRNPA2 was indicated as Green, NSP1 was indicated as Red. Blue is DAPI.


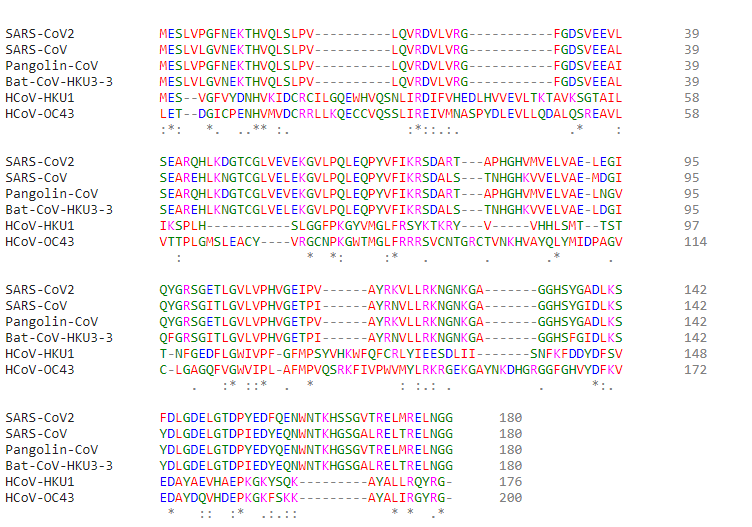


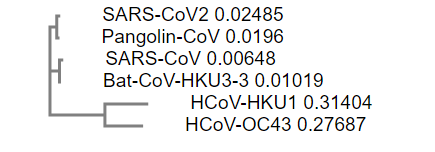


**Supplementary Fig.2** Sequence alignment show NSP1 of HCoV-OC43 and HCoV-HKU display the lowest similarity among other β-coronaviruses, including SARS-CoV, SARS-CoV-2, Pangolin CoV, Bat CoV and HCoV-HKU1. Upper panel, sequence alignment was performed by using CLUSTAL W; lower panel, the phylogenetic tree. The sequence ID: SARS-CoV-2 YP_009742608.1; SARS coronavirus, QJE50588.1; Pangolin coronavirus, QIQ54046.1; Bat SARS coronavirus HKU3-3, AAZ41339.1; Human coronavirus OC43, YP_009924319; Human coronavirus HKU1, YP_460018.1

a


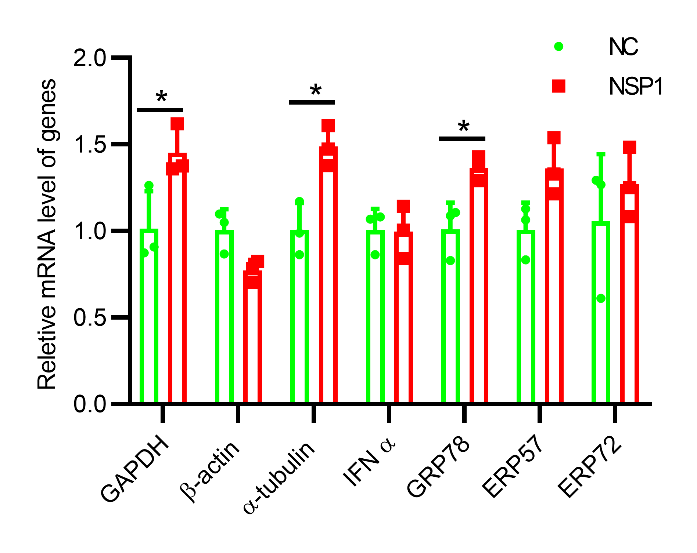


NC

GAPDH


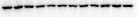


hnRNP A2


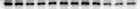


NSP1

NSP2

NSP3

NSP4

NSP5

NSP9

NSP12

NSP13

NSP14

NSP15

ORF8

b

**Supplementary Fig.3**.**Effects of SARS-CoV-2 proteins on host gene expressions** (a)293T cells were transfected with a NSP1-expressing plasmid for 48 hours, then the total cellular RNA was isolated and applied for quantitative RT-PCR (RT-qPCR). Ribosomal 18S RNA was used as internal control, two tail unpaired t test, a *p* value less than 0.05 was considered as statistically significance and marked with a star. (b) The Western Blot result (hnRNP A2/B1) of 293T cells transfected with different viral protein plasmids (NSP1, NSP2, NSP3, NSP4, NSP5, NSP9, NSP12, NSP13, NSP14, NSP15, ORF8) after 48 hours. GAPDH was loaded as internal control.

**Methods and Materials**

**Cell Culture and Transfection**

Human muscle rhabdomyosarcoma (RD) cell (ATCC CCL-136), Vero cell (ATCC CCL-81), and HEK 293T cell (ATCC CRL-3216) were maintained in Dulbecco’s modified Eagle’s medium (DMEM) containing 10% fetal bovine serum (FBS) with 100U/ml penicillin and 100 μg/ml streptomycin, and cultured in an incubator in condition of 37°C and 5% CO2. Plasmids were transfected in HEK 293T cell using PEI, while transfected in RD cell using Lipofectamine 2000 (Thermo Fisher, USA).

**Plasmid**

The plasmids of SARS-Cov2 viral proteins were kindly constructed by Dr. PH Wang’s Lab (Zheng et al., 2020). Vectors are either pCAG or pcDNA6B.

**Immunofluorescence microscopy**

RD cells were transinfected with plasmids of NSP1, NSP2, NSP5, NSP8 and NSP12. Forty-eight hours post-infection, the monolayer was fixed with 4% of paraformaldehyde (PFA). Cells were blocked with 5% BSA for 30 min at room temperature. Primary antibodies were incubated overnight at 4°C, after which the monolayer was washed three times with PBST. The samples were then incubated with secondary antibodies for 2 h at room temperature. After being washed with PBST for 3 times, the samples were treated with DAPI for 5 min at room temperature. The monolayer was then washed three times with PBST. Cells were then visualized using an inverted fluorescent microscope (Nikon Eclipse Ti).

**Western blotting**

Cells were lysed in Nonidet-P40 (NP-40) buffer (150 mM sodium chloride, 1.0% NP-40, 50 mM Tris, pH 8.0, 1×Roche protease inhibitor cocktail) with occasional vortex. The cell lysates were then centrifuged to remove debris at 14,000 rpm for 20 min at 4°C. The concentration of proteins in the lysates was determined by Bradford assay (Bio-Rad). Equal amounts of total protein for each sample was loaded and separated by 8%-12% SDS-PAGE and then transferred onto polyvinylidene difluoride (PVDF) membranes (Amersham Biosciences). Membranes were blocked with 5% Bull Serum Albumin (BSA) in TBST (20 mM Tris-HCl, pH 7.4, 150 mM NaCl, 0.1% Tween 20) for 1 h and incubated with specific antibodies. Beta-actin or GAPDH was served as the loading control. Target proteins were detected with corresponding secondary antibodies (Santa Cruz Biotechnology, USA), visualized with a C600 western blot imaging system from Azure Biosystems.

**RNA interference**

RNA interference was carried out using siRNA purchased from Genepharma (ShangHai, China). Sequences are as follows:

si-hnRNP-A1 GCUCUUCAUUGGAGGGUUG CAACCCTCCAATGAAGAGC
si-hnRNP-A2 CAGAAGAAAGUUUGAGGAACUACUAUAGUAGUUCCUCAAA CUUUCUUCUG

Transfection of siRNA was performed according to the manufacturer’s instructions. Cells at 50% confluence were transfected with 40 nM siRNA using the Hiprefect.

**Quantitative Real-Time Polymerase Chain Reaction**

1 μg of total RNA was subjected to reverse transcription using PrimeScript™ Reverse Transcription System (TaKaRa, Japan). The real-time PCR was carried out in the Applied Biosystems QuantStudio™ 3 Real-Time PCR Systems (96 well plate) with TB Green Premix Ex Taq II (TaKaRa, Japan) using the following program: 95°C for 30s followed by 40 cycles of 95°C for 5s and 60°C for 30s. Three independent experiments with triplicate samples were performed. Target gene’s RNA transcriptional level was normalized to GAPDH in the same sample and results were calculated using delta CCT method. The primers used in this study were:

GAPDH-F: GTCTCCTCTGACTTCAACAGCG

GAPDH-R: ACCACCCTGTTGCTGTAGCCAA

IFN beta-F: CTTGGATTCCTACAAAGAAGCAGC

IFN beta-R: TCCTCCTTCTGGAACTGCTGCA

IFN alpha1-F: TAGACAAATTCTGCACCGAAC

IFN alpha1-R: AGATGGAGTCCICATTCATC

h-CovOC43-F: GGCTTATGTGGCCCCTTACT

h-CovOC43-R: GGCAAATCTGCCCAAGAATA

18sRNA-F: CTACCACATCCAAGGAAGCA

18sRNA-R: TTTTTCGTCACTACCTCCCCG

h-aTubulin-F: GCCTGGACCACAAGTTTGAC

h-aTubulin-R: TGAAATTCTGGGAGCATGAC

hACTB-F: GTTGCTATCCAGGCTGTGCT

hACTB-R: GAGGGCATACCCCTCGTAGA

hIL6-F: AGACAGCCACTCACCTCTTCAG

hIL6-R: TTCTGCCAGTGCCTCTTTGCTG

hTNF-alpha-F CTCTTCTGCCTGCTGCACTTTG

hTNF-alpha-R ATGGGCTACAGGCTTGTCACTC

ERP57-F: GTGCTAGAACTCACGGACGA

ERP57-R: GCTGCAGCTTCATACTCAGG

ERP72-F: TCCTGCTCCTGCTGCTCTTGG

ERP72-R: TCCTCCTCCTCCTCCTCTTCATCC

GRP78-F: CAAGAACCAGCTCACCTCCAA

GRP78-R: ACCACCTTGAACGGCAAGAAC

ISG56-F: TCCCCTAAGGCAGGCTGTC

ISG56-R: GACATGTTGGCTAGAGCTTCTTC

OAS1-F: TCCACCTGCTTCACAGAACTACA

OAS1-R: TGGGCTGTGTTGAAATGTGTTT

RNAaseL-F: AAGGCTGTTCAAGAACTACACTTG

RNAaseL-R: TGGATCTCCAGCCCACTTGATG

**Immunoprecipitation**

Cell lysates were prepared and precleared for 1 h at 4°C with protein A/G-agarose (Santa Cruz Biotechnology). Anti-hnRNP A2/B1 and anti-Flag were incubated with cell lysates. Immune complexes were collected after overnight incubation at 4°C. After washing three times with lysis buffer, immune complexes were resolved by SDS–polyacrylamide gel electrophoresis (PAGE) and transferred to nitrocellulose membranes (Bio-Rad).

**Reference**

Zheng Y, Zhuang MW, Han L, Zhang J, Nan ML, Zhan P, Kang D, Liu X, Gao C, Wang PH. Severe acute respiratory syndrome coronavirus 2 (SARS-CoV-2) membrane (M) protein inhibits type I and III interferon production by targeting RIG-I/MDA-5 signaling. *Signal Transduct Target Ther*. **2020** Dec 28;5(1):299. doi: 10.1038/s41392-020-00438-7.
